# Impact of right ventricular end systolic volume and mitral regurgitation on survival in patients with severe ischemic cardiomyopathy

**DOI:** 10.1186/1532-429X-15-S1-P252

**Published:** 2013-01-30

**Authors:** Deborah Kwon, Zoran B Popovic, Milind Y Desai, Scott D Flamm, Thomas Marwick, Rory Hachamovitch

**Affiliations:** 1 Cleveland Clinic, Cleveland, OH, USA; 2 Cardiovascular Imaging, Menzies Research Institute, Hobart, TAS, Australia

## Background

Mitral regurgitation (MR) and right ventricular dysfunction have been shown to be an independent predictors of mortality in patients with severe ischemic cardiomyopathy (ICM). However, it is unclear how right ventricular end systolic volume index (RVESVi) modifies risk in patients with ischemic mitral regurgitation. We sought to assess impact of RVESVi, MR, and the interaction of these variables on outcomes in patients with ICM.

## Methods

450 patients with > 70% stenosis in ≥1 epicardial coronary artery (75% men, median age 63 years, median LV ejection fraction (EF) 22 %, median ESVi 106ml, median scar % of 29% ) underwent delayed hyperenhancement-MRI (Siemens 1.5-T scanner, Erlangen, Germany) between 2002-2006. CMR evaluation included long and short axis assessment of LV and RV function on balanced steady state free precession images along with assessment of LV and RV myocardial scar (on phase-sensitive inversion recovery DHE-CMR sequence ~ 10-20 minutes after injection of 0.2 mmol/kg of Gadolinium dimenglumine). Scar was identified as regions of interest > 2 SD above normal myocardium. Cox proportional hazards survival modeling, using a primary end-point of all-cause mortality, was used to risk-adjust comparisons. MR severity was determined by echocardiography and assessed by width of the vena contracta. Cox proportional hazards survival modeling, using a primary end-point of all-cause mortality, was used to risk-adjust comparisons.

## Results

**Figure 1:**
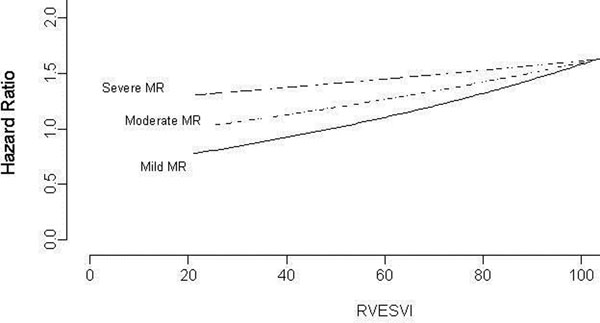


Over a follow-up of up to 9 years[mean 5.8years], 186 deaths occurred. Survival analysis revealed that after adjusting for age, gender, subsequent CABG, scar burden, and presence of ICD, RVESVi (χ2 16.64, p = 0.0002), MR (χ2 14.17, p=0.0008), and the interaction RVESVi Χ MR (χ2 8.17, p = 0.0043) were independent predictors of mortality. Increasing RVESVi mitigated the risk associated with increasing MR. On the other hand, increasing MR resulted in increasing risk in patients with lower RVEVi (Figure 1).

## Conclusions

RVESVi and MR are both powerful independent predictors of mortality; however, increasing RVESVi impacts the risk associated with ischemic MR in patients with severe ICM.

## Funding

None

